# The Beef Bonanza

**Published:** 1881-01

**Authors:** 


The Beef Bonanza; or, How to Get Rich on the Plains. By Gen. James S. Brisbin, U. S. A. With eight illustrations. Philadelphia: J. B. Lippincott & Co. 1880. 16mo, pp. 222.

The author of the above work has certainly struck a fruitful theme, and one which cannot fail to interest the many who are anxious to learn of the opportunities for wealth offered by the great West. The way to get rich on the Plains does not lie in following directions for hunting and trapping wild animals, but in taking advantage of our great grass-covered areas, by making the tegateska take the place of the tatonka—the tame steer graze over the stamping-ground of the wild buffalo.

The questions so often asked, "Where had I best to settle?" "Where can I buy the cheapest and best land?" "Where will I be safe?" "Where can I raise the best stock?" are in this little work clearly and intelligently answered. Of the various fields for settlement he says:

"I believe Kansas and Iowa are the best unsettled farming States; Nebraska is the best State for farming and stock raising combined; Colorado is the best State for sheep growing, farming, and mining; Wyoming is the best Territory for cattle growing alone; Montana is the best Territory for cattle growing and mining."

Twelve years in the saddle, spent in traversing the most of the territory between the Missouri and Pacific Coast, have given Gen. Brisbin authority to say what he has to say upon the capacity of the great West for stock raising. He argues that it is no use for the young men of the present day in the East to bemoan the high price of land and the few chances of wealth at hand, when better and almost unbounded facilities are offered beyond the Missouri. "*Now* is the time; in a few decades is will be too late."

Under the heading "Cattle-growing out West" he gives a vigorous description of "The Land to the West of us—Great Lands and Great Owners—Estimated Fortunes—The Money to be Made—Millions in Beef," &c.

Under the head of "Sheep-Farming in The West," he describes The Raising of Sheep—Where it is Done—The Wool Crop of the World—Sheep-

Walks of the Plains—A Fortune in Clip—How Ranches are Managed—Kind of Ranch to Select—Profits of Sheep-growing," &c.

"Horse-raising in the West" explains "Who the Horse-raisers are—How they Manage their Herds—Profits of Horse Raising under Favorable Conditions—Horse Notes," &c.

Our great natural grazing ground, he says, comprises 1,650,000 square miles, and here, along the banks of its lacework of streams, are located the great beef bonanza of the present and future.

Numerical totals give but a small and faint idea of future possibilities in the raising of cattle, sheep, and horses, as well as dairying, to which the West invites us. The number of people is increasing faster than the number of cattle. Thus the sure home market is growing, while the whole world without clamors for cheap beef and mutton. "For ten years at least yet, the stock growers need have no fear of overstocking the beef market."

From the general to the particular the author goes with the bent of a practised mind, giving faithful pictures of life on the ranches and ranges, and clear calculations of the profits to be made. The latter he bases on the actual experience of the settlers.

He is enthusiastic in his beliefs, lively in his descriptions, but cautious in his estimates; so much so that one is scarcely aware what a purely commercial book he is reading until he pauses to think how much he has read in other books about the Plains without getting any practical information. More than ever, in his belief, wealth, freedom and growth are the rewards of well-applied industry in the great West, and he blesses Horace Greeley in his grave for the advice he gave so often, "Go West, young man, go West."

